# Reduced L-Carnitine Transport in Aortic Endothelial Cells from Spontaneously Hypertensive Rats

**DOI:** 10.1371/journal.pone.0090339

**Published:** 2014-02-28

**Authors:** Rocío Salsoso, Enrique Guzmán-Gutiérrez, Pablo Arroyo, Carlos Salomón, Sonia Zambrano, María Victoria Ruiz-Armenta, Antonio Jesús Blanca, Fabián Pardo, Andrea Leiva, Alfonso Mate, Luis Sobrevia, Carmen María Vázquez

**Affiliations:** 1 Cellular and Molecular Physiology Laboratory (CMPL), Division of Obstetrics and Gynaecology, School of Medicine, Faculty of Medicine, Pontificia Universidad Católica de Chile, Santiago, Chile; 2 Department of Physiology, Faculty of Pharmacy, Universidad de Sevilla, Sevilla, Spain; 3 University of Queensland Centre for Clinical Research (UQCCR), Faculty of Medicine and Biomedical Sciences, University of Queensland, Herston, Queensland, Australia; Medical College of Wisconsin, United States of America

## Abstract

Impaired L-carnitine uptake correlates with higher blood pressure in adult men, and L-carnitine restores endothelial function in aortic rings from spontaneously hypertensive rat (SHR). Thus, endothelial dysfunction in hypertension could result from lower L-carnitine transport in this cell type. L-Carnitine transport is mainly mediated by novel organic cation transporters 1 (Octn1, Na^+^-independent) and 2 (Octn2, Na^+^-dependent); however, their kinetic properties and potential consequences in hypertension are unknown. We hypothesize that L-carnitine transport kinetic properties will be altered in aortic endothelium from spontaneously hypertensive rats (SHR). L-Carnitine transport was measured at different extracellular pH (pH_o_ 5.5–8.5) in the absence or presence of sodium in rat aortic endothelial cells (RAECs) from non-hypertensive Wistar-Kyoto (WKY) rats and SHR. Octn1 and Octn2 mRNA relative expression was also determined. Dilation of endothelium-intact or denuded aortic rings in response to calcitonine gene related peptide (CGRP, 0.1–100 nmol/L) was measured (myography) in the absence or presence of L-carnitine. Total L-carnitine transport was lower in cells from SHR compared with WKY rats, an effect due to reduced Na^+^-dependent (Na^+^ *_dep_*) compared with Na^+^-independent (Na^+^ *_indep_*) transport components. Saturable L-carnitine transport kinetics show maximal velocity (*V*_max_), without changes in apparent *K*_m_ for Na^+^ *_indep_* transport in SHR compared with WKY rats. Total and Na^+^ *_dep_* component of transport were increased, but Na^+^ *_indep_* transport was reduced by extracellular alkalization in WKY rats. However, alkalization reduced total and Na^+^ *_indep_* transport in cells from SHR. Octn2 mRNA was higher than Octn-1 mRNA expression in cells from both conditions. Dilation of artery rings in response to CGRP was reduced in vessels from SHR compared with WKY rats. CGRP effect was endothelium-dependent and restored by L-carnitine. All together these results suggest that reduced L-carnitine transport (likely via Na^+^-dependent Octn2) could limit this compound's potential beneficial effects in RAECs from SHR.

## Introduction

Essential hypertension is characterized by high blood pressure without an identifiable primary cause [1], [2]. Oral administration of L-carnitine, a natural amino acidic compound, in patients with hypertension resulted in an improvement of arterial blood pressure, thus suggesting a beneficial vascular role for this compound in these subjects [3], [4]. Along with the well-described role of L-carnitine on fatty acid mitochondrial metabolism [5], [6], L-carnitine also increases the metabolic activity in human vascular endothelium [7], [8], and improves the bioavailability of nitric oxide (NO) in rat aorta [9] and in fetal lamb pulmonary vasculature [10]. Other studies show that L-carnitine supplementation in healthy subjects improved postprandial flow-mediated dilation after a high-fat meal [11]. Taken together, all these findings suggest that the transport of L-carnitine into the endothelial cells could be an essential, limiting step of its potential beneficial biological effects in hypertension.

The uptake of L-carnitine is mediated by novel organic cation transporters (OCTNs) of which at least three isotypes (rOctn1, rOctn2 and rOctn3) are expressed in rats [12]. Octn1- an Octn3-mediated L-carnitine transport is independent of sodium (Na^+^) with apparent Michaelis-Menten (*K*_m_) values in the range of 2–200 µmol/L [13]–[15] and 3–6 µmol/L [16], [17], respectively. On the contrary, Octn2-mediated transport is Na^+^-dependent with apparent *K*_m_ between 2–20 µmol/L [16], [18]–[23]. Studies performed in spontaneously hypertensive rat (SHR) show that L-carnitine restores endothelial function in preparations of aortic rings [24], [25], and ameliorates the high-systolic arterial blood pressure exhibited by hypertensive animals [5], [8], [26]. Interestingly, there are no reports addressing the properties of L-carnitine transport in endothelial cells from SHR. Thus, we hypothesize that the activity of OCTNs-mediated membrane transport of L-carnitine by the aortic endothelium is reduced in these hypertensive animals.

The results of this study show that aorta endothelial cells from SHR exhibit reduced maximal L-carnitine transport capacity compared with cells from non-hypertensive animals. Transport of L-carnitine was saturable and mediated by a larger Na^+^-independent, Octn1-like compared with a Na^+^-dependent, Octn2-like transport activity in these cells from SHR. However, similar Na^+^-dependent and Na^+^-independent transport components were found in non-hypertensive rats. It is suggested that the observed endothelial dysfunction in SHR could be due to reduced Na^+^-dependent transport of L-carnitine, which could limit the potential beneficial effects of this compound in the endothelial function in hypertension.

## Methods

### Ethics statement and animals

This investigation strictly conforms to the principles outlined in the European Union Guidelines on the protection of animals used for scientific purposes (DIRECTIVE 2010/63/EU of the European Parliament and of the Council). Protocols were approved by the Committee on the Ethics of Animal Experiments of the University of Sevilla (Spain). Normotensive male Wistar-Kyoto (WKY) and spontaneously hypertensive rats (SHR) aged 8 weeks were obtained from the French Animal Production Center, JANVIER S.A.S. (Saint Berthevin Cedex, France). Rats were housed at a temperature of 22–24°C in individual cages and freely fed (*ad libitum*) regular pellet diet (12 mm pellet, Harlan Laboratories, Indianapolis, USA) until they were 10 weeks of age (wa). They were divided into two groups of 15 animals each, i.e., WKY (control) and SHR (hypertensive) animals. Characterization of WKY rats and SHR in terms of the diastolic and systolic blood pressures and body weight was determined at arrival of the animals (8 wa) and at the moment of isolation of aorta endothelial cells (i.e., 10 wa) as reported [26]. Diastolic and systolic blood pressure values were not significantly different (*P*>0.05) at 8 wa (diastolic  =  93 ± 3 mmHg, systolic  =  123 ± 6 mmHg) compared with 10 wa (diastolic  =  95 ± 2 mmHg, systolic  =  124 ± 5 mmHg) in WKY rats. However, these parameters were elevated (*P*<0.01) in SHR at 8 wa (diastolic  =  190 ± 2 mmHg, systolic  =  233 ± 2 mmHg) and 10 wa (diastolic  =  191 ± 2 mmHg, systolic  =  231 ± 1 mmHg) compared with the corresponding systolic or diastolic values in WKY rats, but were not significantly different (*P*>0.05) between them in SHR.

### Cell culture

Aortas from WKY rats and SHR aged 10 weeks were excised and placed in a petri dish containing phosphate-buffered saline (PBS) solution ((mmol/L) NaCl 130, KCl 2.7, Na_2_HPO_4_ 0.8, KH_2_PO_4_ 1.4 (pH 7.4, 4°C)). The tissue was rinsed by changing PBS until free of any visible blood, and the aorta was stripped of adventicia as reported [27]. Rat aorta endothelial cells (RAECs) were isolated by scraping of rat aortic lumen in the presence of medium 199 (M199) (Gibco Life Technologies, Carlsbad, CA, USA) containing 5 mmol/L D-glucose, 10% new born calf serum, 10% fetal calf serum (FCS) (Gibco), 3.2 mmol/L L-glutamine and 100 U/mL penicillin-streptomycin (primary culture medium, PCM) (Gibco), and cultured up to passage 3 (37°C, 5% CO_2_). Twenty-four hours prior to experiments the incubation medium was replaced by M199 containing 2% sera after two rinses with 200 µL in PBS (37°C). Cells were used in passage 3 for most of the experiments. In addition, in some of transport assays confluent freshly isolated cells (i.e., passage 0) or cells in passages 1 or 2 in culture were also used.

### L-Carnitine transport

Total transport of L-carnitine (*TTC*) was defined as the result of the sum of the Na^+^-dependent (hereafter referred as *^TTC^*Na^+^ *_dep_*) and Na^+^-independent (hereafter referred as *^TTC^*Na^+^ *_indep_*) components plus a nonsaturable, lineal component of transport in the range of L-carnitine used in this study (hereafter referred as *m*•[*Car*], where *m* corresponds to slopes of lineal phases of *TTC* at each L-carnitine concentrations [*Car*]) [28]. The *TTC* (0–80 µmol/L L-carnitine, 3 µCi/mL L-[^3^H]carnitine (NEN, Dreieich, FRG), 30 seconds, 37°C) was measured as previously described for other amino acids in primary cultured endothelium [28]. Briefly, *TTC* assays were performed in a Na^+^-containing Krebs ((mmol/L): NaCl 131, KCl 5.6, NaHCO_3_ 25, NaH_2_PO_4_ 1, Hepes 20, D-glucose 5, CaCl_2_ 2.5, MgCl_2_ 1 (pH 7.4, 37°C)) or in Na^+^-free Krebs solution ((mmol/L) *N*-methyl-D-glucamine (NMDG) 120, KCl 5.6, Hepes 20, D-glucose 5, CaCl_2_ 2.5, MgCl_2_ 1 (pH 7.4, 37°C)).

Initial rate for *TTC*, *^TTC^*Na^+^ *_dep_* and *^TTC^*Na^+^ *_indep_* components was derived from slopes of lineal phases of 20 µmol/L L-carnitine transport. Values for L-carnitine transport were adjusted to the one phase exponential association equation considering the least squares fit:
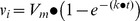
where *v_i_* is initial velocity, *V_m_* is mayor velocity at a given time (*t*) and L-carnitine concentration, and *e* and *k* are constants.

Data for *TTC*, *^TTC^*Na^+^ *_dep_* and *^TTC^*Na^+^ *_indep_* components at initial rates (i.e., linear uptake up to 30 seconds) was adjusted to the Michaelis-Menten hyperbola plus a nonsaturable, lineal component (*m*•[*Car*]) as described [28]:
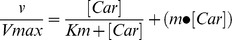
where *v* is the initial reaction velocity relative to the maximal velocity (*V*_max_) and apparent Michaelis-Menten parameter (*K*_m_) of transport at a given L-carnitine concentration ([*Car*]), *m* represents the slope of transport for the range of 0–80 µmol/L L-carnitine and *m*•[*Car*] is the nonsaturable, lineal component of transport in the range of L-carnitine used in this study [28]. Each assay was run in duplicate with transport activity expressed as pmol/µg protein/minute.

After subtracting the *m*•[*Car*] component from *TTC* the remaining transport was defined as total overall saturable transport of L-carnitine (*TSC*). Transport in Na^+^-free Krebs was considered as the Na^+^-independent component of *TSC* (*^TSC^*Na^+^ *_indep_*) and the Na^+^-component (*^TSC^*Na^+^ *_dep_*) was derived from: 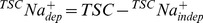



The kinetic parameters *V*_max_ and apparent *K*_m_ for the *TSC*, *^TSC^*Na^+^ *_dep_* or *^TSC^*Na^+^ *_indep_* components were estimating by fitting the data to the single Michaelis-Menten asymptotic hyperbola equation:
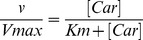



Cells were exposed to PCM 2% sera for a period of 2 hours before the transport assays were performed. Cell viability was assayed by Trypan blue exclusion and was not significantly altered (∼96% of viable cells) in any experimental condition in this study. Rinsing the monolayers with ice-cold Krebs with or without Na^+^ terminated the tracer uptake. Radioactivity in formic acid cell digests was determined by liquid scintillation counting in an automated low activity liquid scintillation analyzer (Tri-Carb 2810TR, PerkinElmer, Santa Clara, CA, USA) with efficiency estimated by converting counts to disintegrations per minute (d.p.m.) [28]. Uptake of L-[^3^H]carnitine was corrected for its extracellular trapping by measuring the accumulation of the non-transportable D-[^14^C]mannitol (1 µCi/mL) (PerkinElmer) in the extracellular space by:
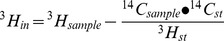
where ^3^ *H_in_* is the L-[^3^H]carnitine associated to the whole cell extracts, ^3^ *H_sample_* and ^14^ *C_sample_* are total L-[^3^H]carnitine and D-[^14^C]mannitol, respectively, for each sample analysed in the scintillation counter, and ^3^ *H_st_* and ^14^ *C_st_* are d.p.m. for standards of L-[^3^H]carnitine and D-[^14^C]mannitol, respectively.

The relative contribution of the hypertension exhibited by SHR to the saturable L-carnitine kinetic parameters (1/*F*) was estimated from the maximal transport capacity (*V*_max_/*K*_m_) values for *TSC* by:
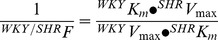
where *^WKY^V*_max_ and *^WKY^K*_m_ are the kinetics parameters for *TSC* in cells from WKY rats, and *^SHR^V*_max_ and *^SHR^K*_m_ are kinetics parameters of transport in cells from SHR. The relative contribution of the *^TSC^*Na^+^ *_dep_* or *^TSC^*Na^+^ *_indep_* components to *TSC* in SHR or WKY rats was estimated from:
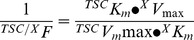
where *X* represents the *^TSC^*Na^+^ *_dep_* or *^TSC^*Na^+^ *_indep_* components for the kinetics parameters *V*_max_ and *K*_m_ of transport compared with *TSC* values. The relative contribution of the *^TSC^*Na^+^ *_dep_* transport compared with the *^TSC^*Na^+^ *_indep_* component to L-carnitine transport in SHR or WKY rats was estimated from:
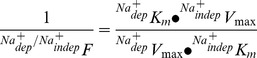



### Extracellular pH dependency

To assay the effect of extracellular pH (pH_o_) on *TSC*, *^TSC^*Na^+^ *_dep_* or *^TSC^*Na^+^ *_indep_* components of L-carnitine transport (20 µmol/L L-carnitine, 3 µCi/mL L-[^3^H]carnitine, 30 seconds, 37°C) the cells were incubated in Na^+^-containing or Na^+^-free Krebs adjusted to a final pH_o_ of 5.5, 6.5, 7.5 or 8.5 as described [20]. The pH_o_ in the Na^+^-containing Krebs solution was adjusted with 1 N HCl or 1 N NaOH, while the Na^+^-free Krebs solution was adjusted with 1 N HCl or 1 N KOH. The pH_o_ values were monitored with a pHmeter (Oakton Instrument, Vernon Hills, IL, USA) and tracer uptake was terminated as above.

### Isolation of total RNA and reverse transcription

Total RNA was isolated using the Trizol reagent (Invitrogen, Carlsbad, CA, USA). RNA quality and integrity were insured by gel visualization and spectrophotometric analysis (OD_260/280_), and RNA concentration was determined at 260 nm. Aliquots (1 µg) of total RNA were reversed transcribed into cDNA as described [28].

### RT-PCR

Experiments were performed using a Light Cycler 480 Detection System (Roche Diagnostic, Barcelona, Spain) in a reaction mix containing 0.5 µmol/L primers and master mix provided in the brilliant SYBR green qPCR Master Mix (Stratagene, La Jolla, CA, USA). SecureStart *Taq* DNA polymerase was activated (15 minutes, 95°C), and assays included a 95°C denaturation (15 seconds), annealing (20 seconds) at 54°C, and extension (10 seconds) at 72°C (rOctn1, rOctn2 and GAPDH). Product melting temperature values were 86°C (rOctn1), 86°C (rOctn2) and 85°C (GAPDH). Oligonucleotide primers: rOctn1 (sense) 5′-TGATAGCCTTCCTGGGCGATTGG-3′, rOctn1 (*anti*-sense) 5′-AAGGAGCCACAGAGAACGCCTAC-3′, rOctn2 (sense) 5′-AGGAGCCCATCAGCACACCCACG-3′, rOctn2 (*anti*-sense) 5′-GACGAAGGACGGACGACAGGTGC-3′, GAPDH (sense) 5′-GCCAAAAGGGTCATCATCTCCGC-3′, GAPDH (*anti*-sense) 5′-GGATGACCTTGCCCACAGCCTTG-3′.

The relative mRNA level in each group was estimated from the 2^−ΔΔCT^ method [29]. Data were analyzed using the Light Cycler 480 SW 1.5 relative quantification (delta-delta-Ct) study software (Roche Diagnostic, Barcelona, Spain) and gene expression levels were normalized to GAPDH and given as relative fold change [30]. The GAPDH mRNA level was not significantly altered (*P*>0.05, n = 15) in all experimental conditions used in this study (not shown).

### Rat aorta reactivity

Ring segments of 2–4 mm in length were dissected from rat aorta in cold (4°C) PBS solution. Vessel rings were mounted in a myograph (610M Multiwire Myograph System, Danish Myo Technology A/S, Denmark) for isometric force measurements in a Krebs physiological solution ((mmol/L): NaCl 118.5, KCl 4.7, NaHCO_3_ 25, MgSO_4_ 1.2, KH_2_PO_4_ 1.2, CaCl_2_ 2.5, D-glucose 5.5, 300 µmol/L L-arginine, pH 7.4). Artery rings were maintained at 37°C and constantly bubbled with a mixture of 95% O_2_/5% CO_2_. The optimal diameter for each vessel was adjusted through the determination of the maximal active response evoked by 62.5 mmol/L KCl [28]. Isometric force was measured in response to calcitonine gene related peptide (CGRP, 0.1–100 nmol/L, 5 minutes) (Peptides International, Inc., KY, USA) in 32.5 mmol/L KCl preconstricted vessels, in the absence or presence of 20 µmol/L L-carnitine (30 minutes). In some artery rings, the endothelium was removed by gentle abrasion of the intimal surface. Successful removal of this cell layer was determined by a reduction in the vasodilatation to CGRP. Changes in isometric tension were recorded using the software LabChart (LabChart 7 for Windows, ADInstruments, Australia) coupled to a PowerLab (PowerLab 8/30 Data Acquisition System, ADIntruments, Australia). The tissue responses are as a percentage of maximal contraction induced by 62.5 mM KCl.

### Statistical analysis

Values are mean ± SEM, where n indicates the number of different cell cultures (3–4 replicates). Data reported in this study describe a normal standard distribution and comparison between two or more than two groups were performed by means of Student's unpaired *t*-test and analysis of variance (2-way ANOVA), respectively. If the ANOVA demonstrated a significant interaction between variables, post hoc analyses were performed by the multiple-comparison Bonferroni correction test. The statistical software GraphPad Instat 3.0b and Graphpad Prism 6.0d (GraphPad Software Inc., San Diego, CA, USA) were used for data analysis. *P*<0.05 was considered statistically significant.

## Results

### Overall transport of L-carnitine

**Figure 1:**
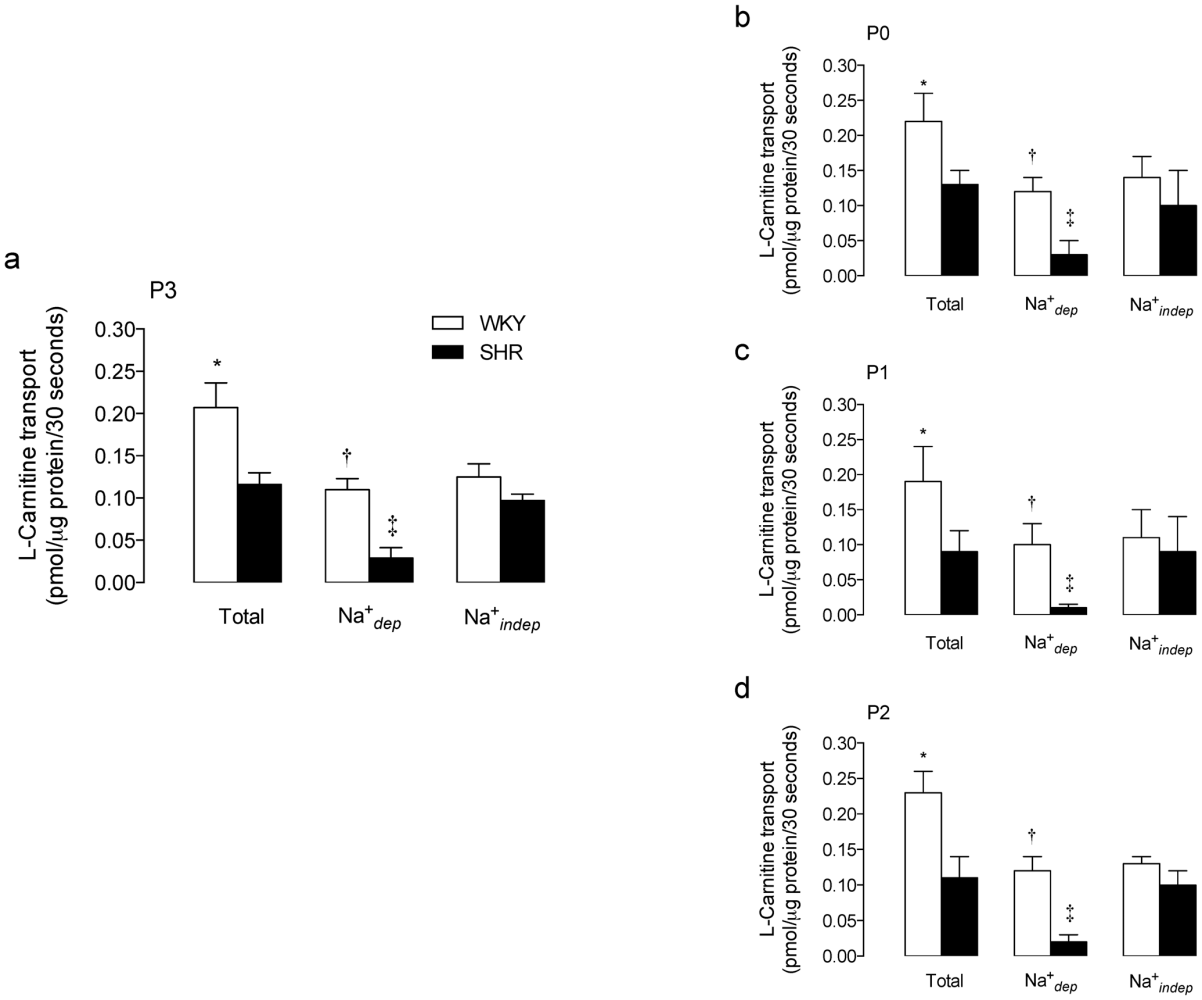
Transport of L-carnitine in RAECs. Total, Na^+^-dependent (Na^+^ *_dep_*) and Na^+^-independent (Na^+^ *_indep_*) L-carnitine transport (20 µmol/L, 3 µCi/mL L-[^3^H]carnitine, 30 seconds, 37°C) in RAECs from WKY rats or SHR. Transport was assayed in RAECs in passage 3 (P3) (a) and compared with cells in passages 0 (P0) (b), 1 (P1) (c) or 2 (P2) (d). *P<0.05 versus all other values, †*P*<0.05 versus corresponding Na^+^ *_dep_* values in SHR, ‡*P*<0.05 versus all other values in SHR. Values are mean ± SEM (n = 7–20).

**Figure 2:**
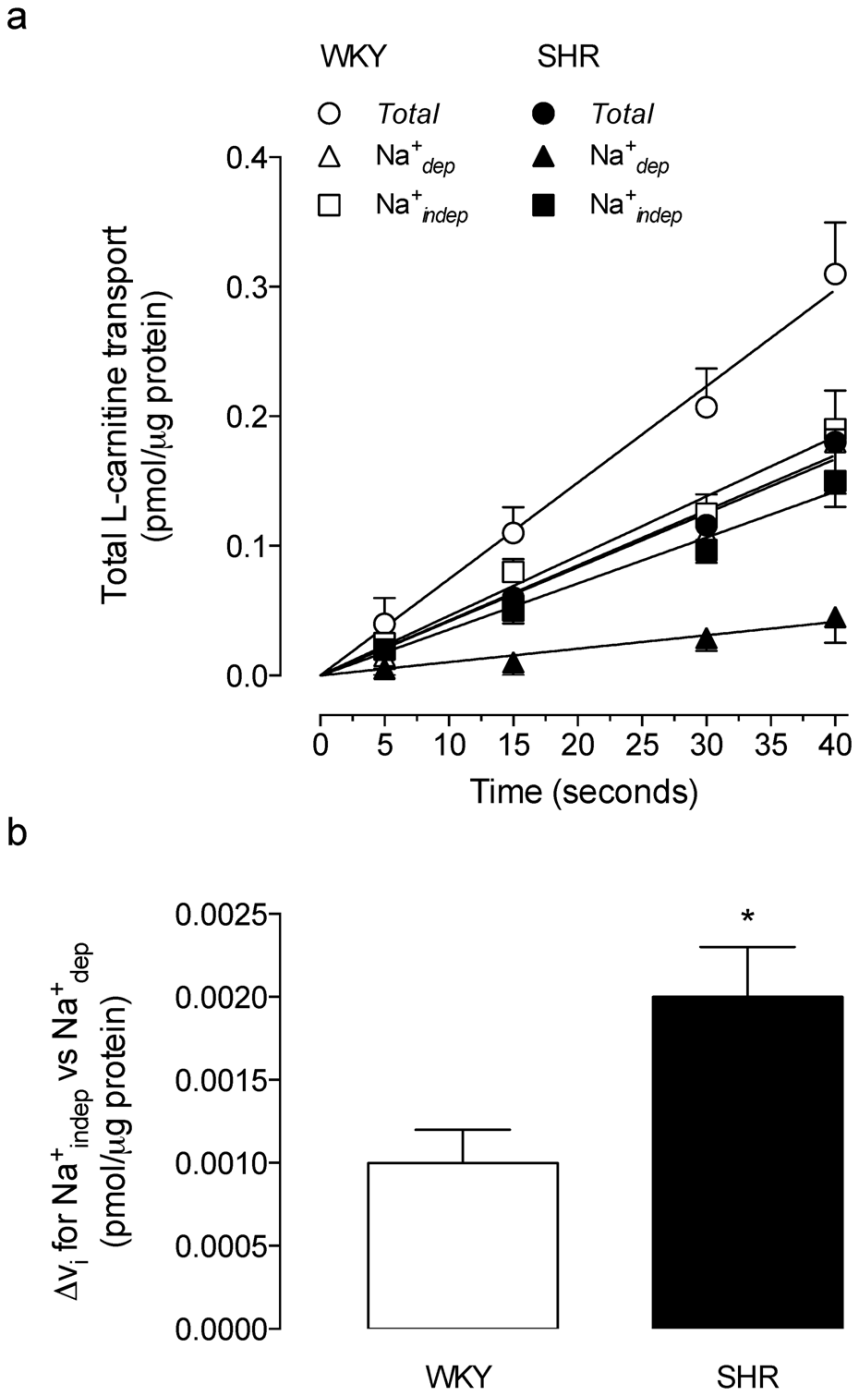
Initial velocities for total transport of L-carnitine. (a) Initial velocity (ν_i_) for total transport of L-carnitine (*Total*), and the Na^+^-dependent (Na^+^ *_dep_*) and Na^+^-independent (Na^+^ *_indep_*) transport components (20 µmol/L L-carnitine, 3 µCi/mL L-[^3^H]carnitine, 37°C) in RAECs cultured from WKY rats or SHR. (b) Difference between the *v*_i_ (Δ*v*_i_) for Na^+^ *_indep_* and Na^+^ *_dep_* components of transport in WKY rats or SHR. *P<0.05 versus WKY. Values are mean ± SEM (n = 15).

The *TTC* for 20 µmol/L L-carnitine was lower (44±8%) in RAECs from SHR compared with WKY rats (Fig. 1a). *TTC* exhibited a *^TTC^*Na^+^ *_dep_* and a *^TTC^*Na^+^ *_indep_* component in cells from both SHR and WKY rats. However, in cells from WKY rats the contribution of the *^TTC^*Na^+^ *_dep_* (52±6%) and *^TTC^*Na^+^ *_indep_* (60±7%) components to the *TTC* were similar (*P*>0.05), whereas, *TTC* in cells from SHR resulted from a major contribution of the *^TTC^*Na^+^ *_indep_* (84±6%) compared with the *^TTC^*Na^+^ *_dep_* (25±5%) component of transport (Fig. 1a). Cells in passage 0 (Fig. 1b), 1 (Fig. 1c) or 2 (Fig. 1d) exhibited similar changes in L-carnitine transport compared with cells in passage 3 (Fig. 1a).

The *TTC* was lineal up to 40 seconds incubation (Fig. 2a) with *v*_i_ values lower in SHR compared with WKY rats (Table 1). In cells from either SHR or WKY rats the *v*_i_ for the *^TTC^*Na^+^ *_indep_* compared with the corresponding *^TTC^*Na^+^ *_dep_* component was higher for 20 µmol/L L-carnitine transport. However, the difference between the *v*_i_ for the *^TTC^*Na^+^ *_indep_* compared with *^TTC^*Na^+^ *_dep_* component was higher (2.1±0.3 fold) in cells from SHR compared with WKY rats (Fig. 2b).

**Table 1:** Kinetic parameters for transport of L-carnitine in rat aortic endothelial cells.

	L-Carnitine transport					
	WKY			SHR		
	Total	Na^+^ *_dep_*	Na^+^ *_indep_*	Total	Na^+^ *_dep_*	Na^+^ *_indep_*
***TTC***						
* v_i_*	0.007±0.0001	0.003±0.002	0.004±0.0003†	0.004±0.0001*	0.001±0.0001*	0.003±0.0001†
* K_D_*	0.0016±0.0002	<10^−13^	0.0013±0.0013	<10^−13^	<10^−15^	<10^−14^
***TSC***						
* V*_max_	0.84±0.2	0.42±0.06	0.46±0.2	0.59±0.07*	0.20±0.03*	0.32±0.08†
* K*_m_	28±9	46±19	21±4	30±8	31±11	22±16
* V*_max_/*K*_m_	0.030±0.008	0.009±0.002	0.022±0.009†	0.020±0.004*	0.006±0.002*	0.015±0.007†

Total (*TTC*) and saturable (*TSC*) transport of L-carnitine and the Na^+^-dependent (Na^+^ *_dep_*) and Na^+^-independent (Na^+^ *_indep_*) components of transport (0–80 µmol/L L-carnitine, 3 µCi/mL L-[^3^H]carnitine, 30 seconds, 37°C, pH 7.4) were measured in cultured (passage 2) rat aortic endothelial cells (RAECs) from normotensive (WKY) or spontaneously hypertensive (SHR) rats as described in Methods. The initial velocity (*v*_i_) was measured for 20 µmol/L L-carnitine up to 30 seconds. *v*_i_, initial velocity (pmol/µg protein/second); *V*_max_, maximal velocity (pmol/µg protein/minute); *K*_m_, apparent Michaelis-Menten constant (µmol/L); *V*_max_/*K*_m_, maximal transport capacity (pmol/µg protein/minute/(µmol/L)); *K*_D_, lineal, non-saturable transport in the range of L-carnitine concentrations used in this study (pmol/µg protein/minute/(µmol/L)). *P<0.05 versus corresponding values in WKY, †*P*<0.05 versus corresponding values for Na^+^ *_dep_* in SHR or WKY rats. Values are mean ± SEM (n = 15).

### L-Carnitine transport kinetics

**Figure 3:**
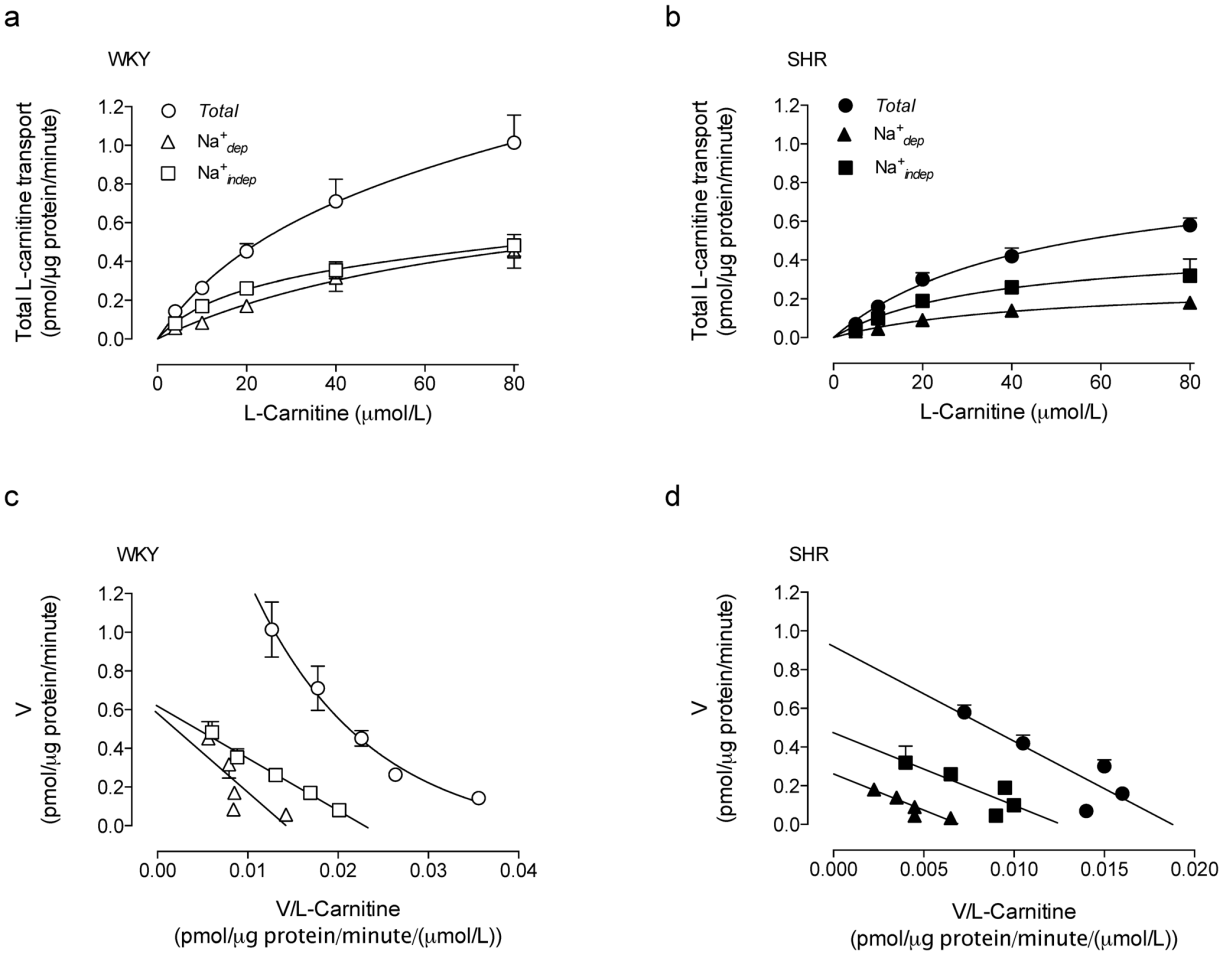
Total transport of L-carnitine kinetics. Total transport of L-carnitine (*Total*), and the Na^+^-dependent (Na^+^ *_dep_*) and Na^+^-independent (Na^+^ *_indep_*) transport components (0–80 µmol/L L-carnitine, 3 µCi/mL L-[^3^H]carnitine, 30 seconds, 37°C) in RAECs cultures from WKY rats (a) or SHR (b). The Eadie-Hofstee plots for transport data in shown for WKY rats (c) and SHR (d) from data in (a) and (b), respectively. Values are mean ± SEM (n = 15).

The *TTC* was semisaturable in RAECs from either SHR or WKY rats (Fig. 3a,b). The *K*_D_ value for *TTC* was similar to that for the *^TTC^*Na^+^ *_indep_* component of transport in cells from WKY rats; however, the *K*_D_ for *^TTC^*Na^+^ *_dep_* transport in cells from these animals and all other *K*_D_ values in SHR were negligible (Table 1). The *^TTC^*Na^+^ *_dep_* and *^TTC^*Na^+^ *_indep_* components derived from *TTC* were saturable in both groups of cells. The Eadie-Hofstee plot of *TTC* data was best fitted by an exponential one phase decay equation resulting in a non-linear plot in cells from WKY rats (Fig. 3c). However, the *^TTC^*Na^+^ *_dep_* and *^TTC^*Na^+^ *_indep_* components derived from *TTC* in WKY rats were linear (Fig. 3c), as were *TTC*, *^TTC^*Na^+^ *_dep_* and *^TTC^*Na^+^ *_indep_* components of transport in SHR (Fig. 3d).

**Figure 4:**
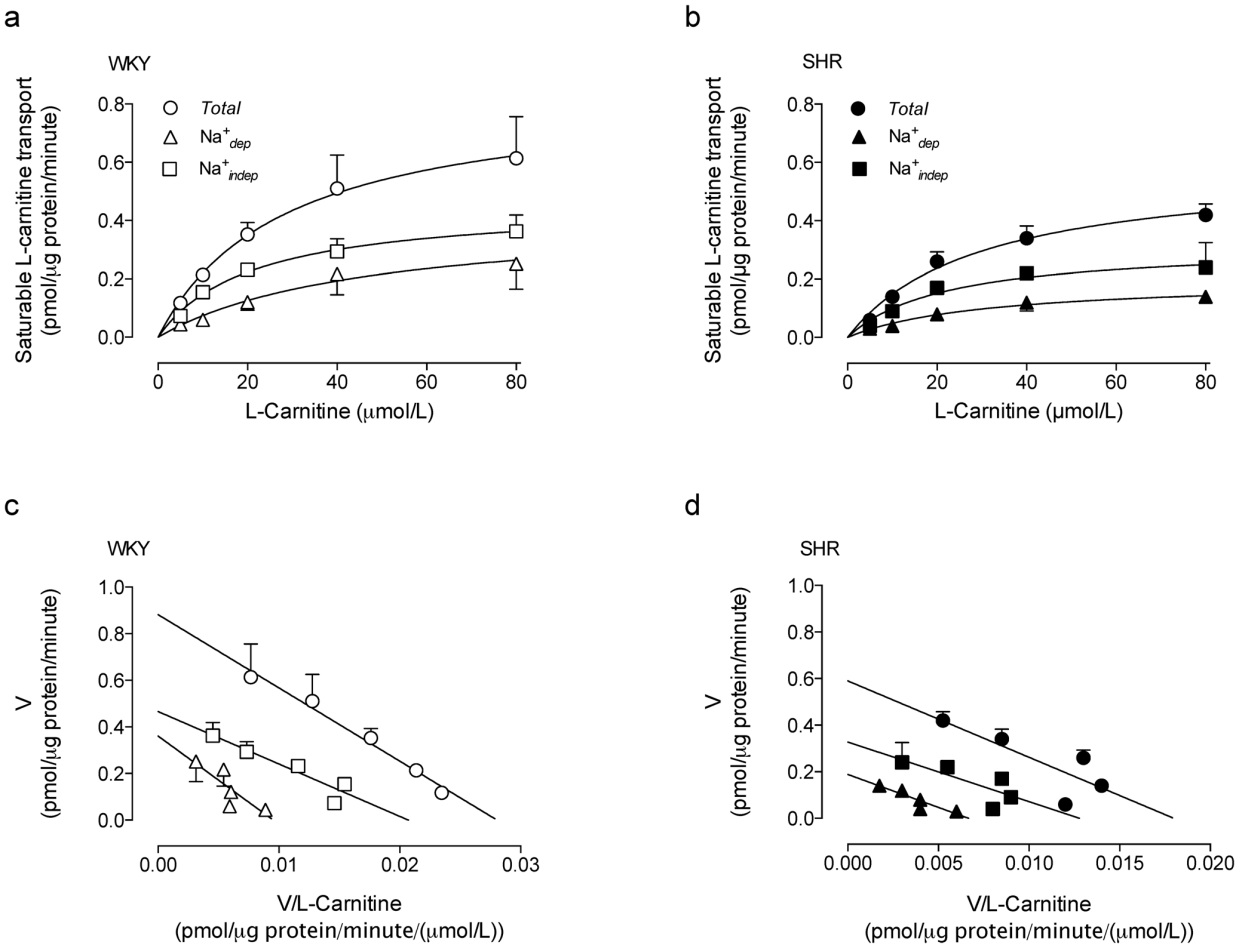
Saturable transport of L-carnitine kinetics. Total saturable transport of L-carnitine (*Total*), and the Na^+^-dependent (Na^+^ *_dep_*) and Na^+^-independent (Na^+^ *_indep_*) transport components (0–80 µmol/L L-carnitine, 3 µCi/mL L-[^3^H]carnitine, 30 seconds, 37°C) in RAECs cultures from WKY rats (a) or SHR (b). The Eadie-Hofstee plots for transport data are shown for WKY rats (c) and SHR (d) from data in (a) and (b), respectively. Values are mean ± SEM (n = 15).

After subtracting the lineal, non-saturable component of transport data (in the range of concentrations used in this study), the saturable transport for each condition was obtained (Fig. 4a,b). Cells from SHR exhibit reduced *V*_max_, but unaltered apparent *K*_m_ for *TSC* compared with cells from WKY rats (Table 1). The *V*_max_ for the *^TSC^*Na^+^ *_dep_*, but not for *^TSC^*Na^+^ *_indep_* components of transport was reduced in cells from SHR compared with the corresponding values in WKY rats. In addition, the *V*_max_ for the *^TSC^*Na^+^ *_indep_* component was higher than *^TSC^*Na^+^ *_dep_* component of transport only in SHR (Table 1). The Eadie-Hofstee plot of saturable transport data was lineal for all experimental conditions (Fig. 4c,d). The *V*_max_/*K*_m_ values for sturable transport and the Na^+^ *_dep_* component were lower in SHR compared with WKY rats, and the value for the *^TSC^*Na^+^ *_dep_* component was lower than the *^TSC^*Na^+^ *_indep_* component of transport in cells from SHR or WKY rats (Table 1).

### Expression of rOctn1 and rOctn2 mRNA

**Figure 5:**
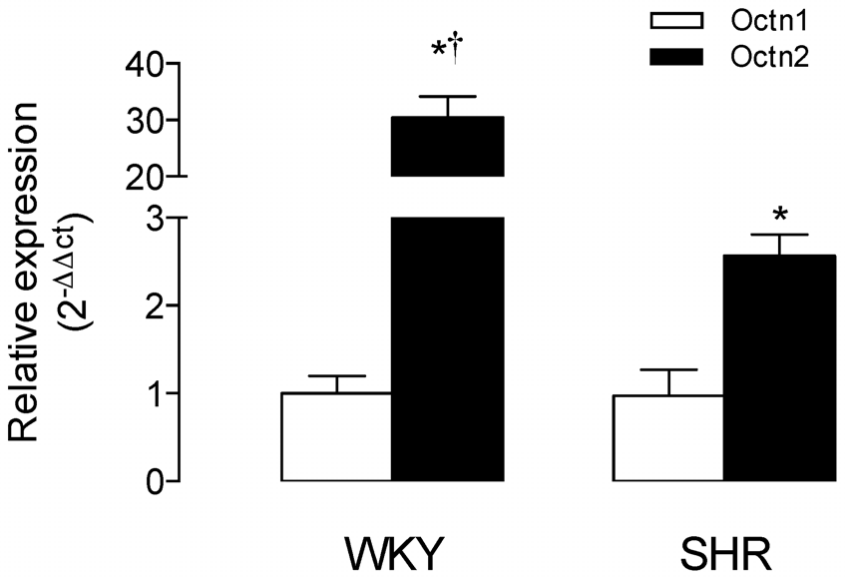
Expression of Octn1 and Octn2. The relative mRNA expression level of Octn1 and Octn2 in RAECs cultures from WKY rats or SHR was estimated from the 2^−ΔΔCT^ method using the Light Cycler® 480 SW 1.5 relative quantification (delta-delta-Ct) study software as described in Methods. Gene expression levels were normalized to GAPDH mRNA level. *P<0.05 versus corresponding values for Octn2. †*P*<0.02 versus corresponding values in SHR. Values are mean ± SEM (n = 15).

The relative expression of rOctn2 was higher than rOctn1 mRNA in RAECs from SHR or WKY rats (Fig. 5). The relative mRNA expression of rOctn2 was largely lower in SHR when compared with WKY; however, no differences were found in mRNA expression of rOctn1 between cells from these animals.

### Extracellular pH dependency on saturable L-carnitine transport

**Figure 6:**
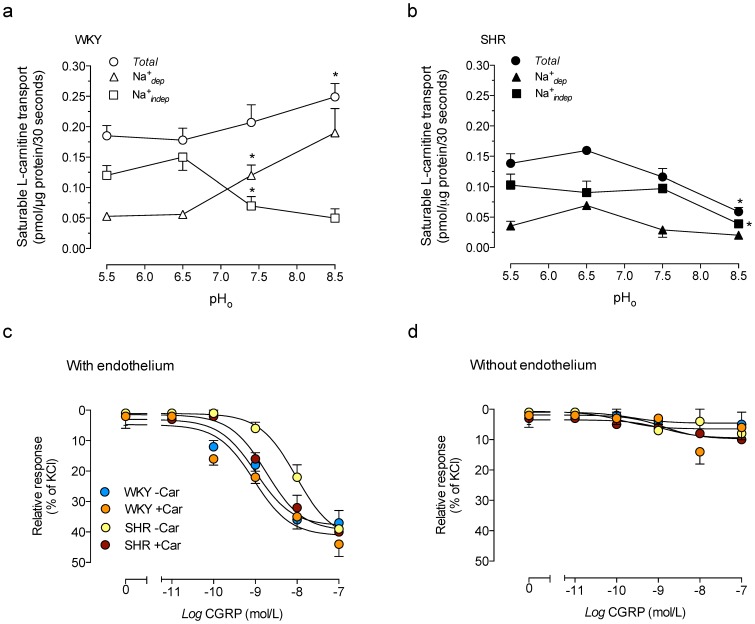
Effect of extracellular pH on saturable transport of L-carnitine, and rat aorta reactivity. Total saturable transport of L-carnitine (*Total*), and the Na^+^-dependent (Na^+^ *_dep_*) and Na^+^-independent (Na^+^ *_indep_*) transport components (20 µmol/L L-carnitine, 3 µCi/mL L-[^3^H]carnitine, 30 seconds, 37°C) in RAECs cultures from WKY rats (a) or SHR (b) exposed to culture medium with the pH adjusted to different values. (c) Relaxation of 32.5 mmol/L KCl preconstricted endothelium-intact aortic vessel rings (With endothelium) from WKY rats or SHR in response to increasing concentrations of calcitonine gene related peptide (CGRP, 5 minutes), in the absence (-Car) or presence (+Car) of 20 µmol/L L-carnitine (30 minutes). (d) Relaxation of endothelium-denuded aortic vessel rings (Without endothelium) to CGRP as in (c). *P<0.05 versus all other values for the corresponding components. Values are mean ± SEM (n = 4–10).

Overall transport of L-carnitine was higher at pH_o_ 8.5 compared with transport at pH_o_ 7.4, but it was unaltered by lower pH_o_ values in cells from WKY rats (Fig. 6a). The *^TSC^*Na^+^ *_indep_* component of transport at pH_o_ 7.4 was higher than at pH_o_ 8.5, but lower than at acidic pH_o_ values. However, the *^TSC^*Na^+^ *_dep_* component of transport at pH_o_ 7.4 was lower than at alkaline, but higher than at acidic pH_o_. The half-maximal stimulatory effect (*SE*_50_) of a change in the pH_o_ on overall transport was higher than the *SE*_50_ for the *^TSC^*Na^+^ *_indep_* (∼0.26 pH_o_ units of difference) component (Table 2). A similar effect on *TSC* was seen for the half-maximal inhibitory effect (*IE*_50_) of a change in the pH_o_ on the *^TSC^*Na^+^ *_dep_* (∼0.53 pH_o_ units of difference) component of transport in cells from WKY rats (Table 2). In addition, the *SE*_50_ for *^TSC^*Na^+^ *_indep_* was significantly different from the *IE*_50_ for *^TSC^*Na^+^ *_dep_* values (∼0.24 pH_o_ units of difference) in these cells.

**Table 2:** Half-maximal effect of extracelular pH on saturable transport of L-carnitine in RAECs.

	*SE*_50_ (pH_o_ units)	*IE*_50_ (pH_o_ units)
WKY		
Total	7.73±0.25	*ni*
Na^+^ *_dep_*	7.47±0.11*	*ni*
Na^+^ *_indep_*	*ns*	7.23±0.14
SHR		
Total	*ns*	7.95±0.13
Na^+^ *_dep_*	*ns*	*ni*
Na^+^ *_indep_*	*ns*	8.10±0.09

Total transport of L-carnitine, and the Na^+^-dependent (Na^+^ *_dep_*) and Na^+^-independent (Na^+^ *_indep_*) transport components (20 µmol/L L-carnitine, 3 µCi/mL L-[^3^H]carnitine, 30 seconds, 37°C) in RAECs cultures from WKY rats or SHR exposed to culture medium with the pH adjusted to different values (extracellular pH (pH_o_) 5.5–8.5) as described in Methods. The stimulatory (*SE*_50_) or inhibitory (*IE*_50_) effect of pH_o_ on transport was calculated. *ni*, not inhibited; *ns*, not stimulated. *P<0.05 versus Total in WKY rats. Values are mean ± SEM (n = 12).

In RAECs from SHR, the *TSC* and the *^TSC^*Na^+^ *_indep_* component of transport were lower at alkaline pH_o_ compared with transport at pH_o_ 7.4 (Fig. 6b). However, the *^TSC^*Na^+^ *_dep_* component of saturable transport was unaltered compared with values at pH_o_ 7.4 in these cell types. The *IE*_50_ values for overall and the *^TSC^*Na^+^ *_indep_* component of L-carnitine transport (∼0.05 pH_o_ units of difference) were not significantly different in cells from SHR (Table 2). However, the alkalization required to reduce the overall transport in cells from SHR was higher (∼0.22 pH_o_ units of difference) compared with the alkalization required to increase the transport in cells from WKY rats. In addition, the alkalization required to reduce the *^TSC^*Na^+^ *_indep_* component of transport in cells from SHR was higher (∼0.93 pH_o_ units of difference) compared with cells from WKY rats.

### Rat aorta reactivity

CGRP caused dilation of preconstricted aortic rings in both group of animals (Fig. 6c). However, the half-maximal vasodilation (*EC*_50_) caused by CGRP was lower in vessels from SHR (*EC*_50_  =  9.5±0.3 nmol/L) compared with WKY (*EC*_50_  =  1.0±0.2 nmol/L) rats. Supplementation with L-carnitine caused a reduction of the *EC*_50_ for CGRP in vessels from SHR (*EC*_50_  =  1.9±0.3 nmol/L), but did not alter (*P*>0.05) this parameter in vessels from WKY rats (*EC*_50_  =  0.9±0.1 nmol/L). CGRP was ineffective in endothelium-denuded rat aortic rings (Fig. 6d).

## Discussion

We have characterized the kinetics of L-carnitine transport in primary cultures of rat aortic endothelial cells (RAECs) from non-hypertensive WKY rats and contrasted this information with cells from spontaneously hypertensive rats (SHR). Total overall transport of L-carnitine (*TTC*) was mediated by Na^+^-dependent (*^TTC^*Na^+^ *_dep_*) and Na^+^-independent (*^TTC^*Na^+^ *_indep_*) components increased by a lineal, nonsaturable mediated transport. A reduced initial velocity for the *TTC* and *^TTC^*Na^+^ *_dep_*, but not the *^TTC^*Na^+^ *_indep_* component was found in cells from SHR compared with non-hypertensive WKY rats. The kinetics assays for saturable overall transport of this amino acid (*TSC*) show that maximal transport capacity (*V*_max_/*K*_m_) for L-carnitine is lower in cells from SHR compared with WKY rats, a finding paralleled by reduced *V*_max_/*K*_m_ for the *^TSC^*Na^+^ *_dep_*, but not the *^TSC^*Na^+^ *_indep_* component. A differential dependency of pH_o_ for *TSC*, *^TSC^*Na^+^ *_dep_* and *^TSC^*Na^+^ *_indep_* transport was seen in cells from SHR compared with WKY rats. These results are the first demonstration that RAECs from SHR exhibit a phenotype characterized by reduced L-carnitine transport compared with cells from non-hypertensive rats. These results are potentially useful for a better understanding of the membrane transport mechanisms of L-carnitine in RAECs from non-hypertensive WKY rats and SHR. Interestingly, a reduced reactivity to an endothelium dependent vasodilator of the aortic rings from SHR compared with WKY rats was seen, an effect that was improved by supplementation of these vessels *in vitro* with L-carnitine. However, vasodilation was absent in endothelium-denuded aortic ring preparations. It is suggested that a reduced uptake of L-carnitine by the endothelium could counteract the reported beneficial vascular effects of L-carnitine supplementation in subjects with hypertension.

### L-Carnitine transport in normotensive rats

Several reports describe that the natural amino acid L-carnitine could act by improving the high arterial blood pressure in patients with essential hypertension [3], [4] and in animal models of hypertension [5], [26]. These results complement the possibility that in hypertension the membrane transport of this amino acid is reduced, a phenomenon that is supported by studies showing a correlation between increased L-carnitine plasma level and higher blood pressure in adult men [31]. Plasma membrane transport of L-carnitine is mediated by OCTNs in mammalian cells, and the isotypes Octn1, Octn2 and Octn3 have been cloned from rats [12], [32]. Our results show that RAECs exhibit *TTC* mediated by at least three different components (Na^+^-dependent (*^TTC^*Na^+^ *_dep_*), Na^+^-independent (*^TTC^*Na^+^ *_indep_*) and lineal, nonsaturable transport) in the range up to 80 µmol/L L-carnitine. These findings agree with the basic characteristics described for Octn-like transport of this amino acid in other cell types [33]–[35]. Since the Eadie-Hofstee representation of the *TTC* data was not lineal, it is likely that at least two or more transport systems acting in parallel [36], [37] will account for L-carnitine transport in RAECs from non-hypertensive rats. In addition, since the *^TTC^*Na^+^ *_dep_* and *^TTC^*Na^+^ *_indep_* components of transport were lineal in the Eadie-Hofstee plot, either a single transport system or two or more transport systems with similar kinetic parameters acting in parallel mediate the Na^+^-dependent and the Na^+^-independent L-carnitine transport in this cell type.

Our results also show that cells from WKY rats exhibit a *TSC* resulting from a pronounced differential contribution of the *^TSC^*Na^+^ *_dep_* and the *^TSC^*Na^+^ *_indep_* components when the relative *V*_max_/*K*_m_ for these components were compared. The relative contribution of the *V*_max_/*K*_m_ for the *^TSC^*Na^+^ *_dep_* component to *TSC* is lower (∼30%) (from 1/^TSC/Na+-dep^ *F*  =  0.30) compared with the contribution accounted by the *^TSC^*Na^+^ *_indep_* component (∼73%) (1/^TSC/Na+-indep^ *F*  =  0.73). Thus, a Na^+^ *_indep_* component of L-carnitine transport predominates in RAECs from non-hypertensive WKY rats.

Octn1 is widely expressed in several tissues, including microvascular endothelium from human heart [38], and mediates L-carnitine transport via a Na^+^-independent mechanism [34], [39] with apparent *K*_m_ values ranging from 2–200 µmol/L [13]–[15]. Since the results of our study show that the apparent *K*_m_ for the *^TSC^*Na^+^ *_dep_* and *^TSC^*Na^+^ *_indep_* components (*K*_m_  =  21–46 µmol/L) was in the range of values described for this membrane transporter isoform in other cell types [35], the possibility that Octn1 was responsible of the Na^+^-independent L-carnitine transport in RAECs from WKY rats is supported. However, Octn2-mediated transport of L-carnitine is described as a Na^+^-dependent transport mechanism with higher affinity (*K*_m_  =  2–20 µmol/L) compared to Octn1 [16], [18]–[22]. Octn2 is also expressed in other types of endothelial cells, including human heart and brain capillaries endothelium [23], [40]; therefore Octn2 could also account for the Na^+^-dependent transport of L-carnitine in RAECs. Interestingly, since the plasma concentration of L-carnitine for WKY rats is reported as 20–36 µmol/L [41] it is likely that the lower affinity transport system Octn1 would play a preferential role compared with Octn2, which is likely to be saturated at physiological L-carnitine plasma concentrations, in maintaining the extracellular physiological concentrations of this amino acid in these animals.

In the present study, both Octn1 and Octn2 mRNA expression was detected in RAECs from WKY rats. Interestingly, Octn2 mRNA relative expression resulted to be ∼31 fold higher compared with Octn1 mRNA in these cells. Since the relative contribution of the *^TSC^*Na^+^ *_dep_* component to the *V*_max_/*K*_m_ for *TSC* was ∼30%, it is likely that not more than ∼10 fold change in Octn2 mRNA expression (estimated from the (Octn2 mRNA/Octn1 mRNA)/(1/^TSC/Na+-dep^ *F*) ratio) could sustain a *^TSC^*Na^+^ *_dep_* component for the *TSC* in RAECs. The remaining transport mediated via a *^TSC^*Na^+^ *_indep_* component (∼73%) could represents the contribution of a Na^+^-independent transport activity derived from Octn1 in this cell type. Interestingly, since the contribution for the Na^+^-dependent Octn2 transport is reported as ∼3 fold higher than the transport detected in the absence of extracellular Na^+^ in other cell types [42], an equivalent fractional contribution for these components to L-carnitine transport in RAECs from WKY rats could be expected. However, the latter seems unlikely in this cell type since the relative contribution of the *^TSC^*Na^+^ *_dep_* component to the *V*_max_/*K*_m_ for *TSC* was ∼30% (1/^TSC/Na+-dep^ *F*  =  0.3) compared with ∼73% (1/^TSC/Na+-indep^ *F*  =  0.73) for the *^TSC^*Na^+^ *_indep_* component. Thus, contrasting with other cell types [35], [42], these findings further support the possibility that the Na^+^ *_indep_* component predominates (∼2.4 fold) compared with the Na^+^ *_dep_* component regarding their contribution to *TSC*. This could be interpreted as a major contribution of Octn1 compared with Octn2 to the saturable transport of L-carnitine in RAECs from WKY rats.

L-Carnitine transport in RAECs from WKY rats was also dependent on the pH_o_, a characteristic well described for Octn1 [13], [32] and Octn2 [43]. Our results show that *TSC* was increased by ∼20% by alkalization of the extracellular medium, an effect resulting from a combined increases in the *^TSC^*Na^+^ *_indep_* component (∼58%) and decreases in the *^TSC^*Na^+^ *_dep_* component (∼29%). This phenomenon could be due to a higher sensitivity to alkalization of the *^TSC^*Na^+^_dep_ [(*TSC SE*_50_) minus (*^TSC^*Na^+^_dep_ *IE*_50_)  =  0.26 units of pH_o_] compared with the *^TSC^*Na^+^_indep_ [(*TSC SE*_50_) minus (*^TSC^*Na^+^_indep_ *IE*_50_)  =  0.50 units of pH_o_] components regarding the change seen in *TSC*. Based in these findings, a higher alkalization-dependent increase in the *TSC* could be reached whether these two transport components were equally altered or whether the *^TSC^*Na^+^ *_indep_* component was unaltered by this environmental condition. Interestingly, the increase of *TSC* caused by a change of 0.6 units of pH_o_ in RAECs was ∼3.3 fold the increase reported for 150 µmol/L tetraethylammonium (TEA) uptake in response to a similar change in pH_o_ units in HEK293 cells expressing the human OCTN1 form [13]. However, contrasting with these results a larger increase in the pH_o_ value (1 unit of pH_o_) reduced overall transport of TEA in these cells [32]. Thus, it is likely that OCTNs-like transport is differentially responsive to the degree of alkalization reached in HEK293 cells. Interestingly, a change in ∼0.4 units of pH_o_ has been shown to increase the activity of other membrane transport systems, such as the sodium/proton exchanger isoform 1 in MDCK cells [44], suggesting that modulation of Octn1/2 by a similar change in the pH_o_ in RAECs agree with what is reported in other cell types. On the other hand, acidification of the extracellular pH_o_ does not alter *TSC*, a net effect that result from a proportional reduced *^TSC^*Na^+^ *_dep_* and increased *^TSC^*Na^+^ *_indep_* transport components. Interestingly the effect of extracellular acidification was similar for both components, suggesting that these components are equally sensitive to acidification in RAECs from WKY rats. Thus, acidification and alkalization of extracellular medium results in a differential modulation of L-carnitine transport in RAECs from non-hypertensive rats.

### Effect of spontaneous hypertension on L-carnitine transport

Cells from SHR exhibit a semisaturable *TTC* unaffected by a lineal, non-saturable component in the range of L-carnitine concentrations used in this study. This data was best fitted to a first-order regression line in an Eadie-Hofstee plot, suggesting that one or more transport systems with apparent *K*_m_ values in the same range could mediate L-carnitine transport in RAECs from SHR. The apparent *K*_m_ values for L-carnitine transport by RAECs in SHR were similar to values detected in non-hypertensive rats, and within the range of the plasma L-carnitine concentration reported in SHR (21–41 µmol/L) [41]. Thus, L-carnitine transport via Octn1/2 could contribute to maintain the physiological plasma concentration of this amino acid in SHR. Our results also show that *TTC* is mainly mediated by a *^TTC^*Na^+^ *_indep_* (∼75%) with a minor contribution of *^TTC^*Na^+^ *_dep_* (∼25%) components. These findings agree with those obtained by contrasting the relative *V*_max_/*K*_m_ for these components with that for *TSC*. Since the relative contribution of the *V*_max_/*K*_m_ for the *^TSC^*Na^+^ *_indep_* component is higher (∼75%) (1/^TSC/Na+-indep^ *F*  =  0.75) compared with the contribution accounted by the *^TSC^*Na^+^ *_dep_* component (∼30%) (1/^TSC/Na+-dep^ *F*  =  0.30), and considering that similar findings were found for the *v*_i_ values for these transport components, it is suggested that RAECs from SHR exhibit saturable transport of L-carnitine where the Na^+^-independent transport predominates by ∼2.5 fold compared with the Na^+^-dependent. This result is ∼2.1 fold higher compared with cells from non-hypertensive rats supporting the possibility that hypertension could associate with a higher requirement of Na^+^-independent transport of L-carnitine via Octn1/2 activity in RAECs. Thus, it is likely that a deficiency in the *^TSC^*Na^+^ *_dep_* component results in RAECs dysfunction in SHR. This would not be explained by a lower *V*_max_/*K*_m_ of the *^TSC^*Na^+^ *_dep_* component, since the relative contribution of this component to *TSC* in these cells was similar to that in cells from non-hypertensive rat ((1/^TSC/Na+-dep^ *F* in WKY rats)/(1/^TSC/Na+-dep^ *F* in SHR  =  1.01). In addition, the relative contribution of the *^TSC^*Na^+^ *_dep_* component compared with the *^TSC^*Na^+^ *_indep_* component to *TSC* in SHR is also similar to non-hypertensive rats ((1/^Na+-indep/Na+-dep^ *F* in WKY rats)/(1/^Na+-indep/Na+-dep^ *F* in SHR  =  1.03). Thus, reduced overall transport of L-carnitine in RAECs from SHR could be mainly due to reduced expression of the Na^+^-dependent Octn2 and in a less extend to a reduced expression of the Na^+^-independent Octn1 membrane transporters. In fact, the Octn2 mRNA expression in cells from SHR is only 2.6 fold compared with Octn1 mRNA expression, a value that is largely minor compared with the 31 fold increase for this mRNA detected in cells from non-hypertensive rats. Thus, a reduced Octn2 expression without alterations in the *V*_max_/*K*_m_ could account for the reduced *^TSC^*Na^+^ *_dep_* component of L-carnitine transport in RAECs from SHR.

Interestingly, as found in cells from non-hypertensive rats, the relative contribution of the *^TSC^*Na^+^ *_dep_* component to the *V*_max_/*K*_m_ for *TSC* was ∼30%. Thus, a proportional change by Octn2 expression to (i.e., ∼1.48 fold) would sustain the *^TSC^*Na^+^ *_dep_* component of the saturable transport activity in RAECs from SHR. Interestingly, this value is ∼85% lower compared with the potential requested change in Octn2 mRNA expression in cells from non-hypertensive rats. Therefore, SHR is a pathological condition that results in lower request of Octn2 mRNA expression compared with RAECs from non-hypertensive rats. The results also show that L-carnitine transport in RAECs from SHR was dependent on the pH_o_, supporting the possibility that transport was mediated by Octn1/2 in this cell type. In this case, alkalization of the extracellular medium resulted in reduced *TSC*, which was due to reduced Na^+^ *_indep_* component. This finding is different from RAECs from non-hypertensive rats, suggesting that alkalization could result in a differential down-regulation of L-carnitine transport in RAECs from SHR compared with non-hypertensive rats. However, since the Na^+^ *_indep_* component of transport was also reduced in cells from non-hypertensive rats, it is likely that sensitivity of this component to a change in the pH_o_ is similar in cells from SHR and WKY rats.

In conclusion, the kinetic parameters of L-carnitine transport in RAECs from SHR and non-hypertensive WKY rats were characterized. The overall saturable transport was mediated by Na^+^ *_indep_* and Na^+^ *_dep_* components, with the latter being crucial in the reduced maximal transport capacity detected in cells from SHR. The kinetic parameters, pH_o_- and Na^+^-dependency of transport suggest that Octn1 and Octn2 are likely responsible for membrane transport of L-carnitine in endothelial cells from the aorta of SHR and WKY rats. These results are the first characterizing the kinetic parameters for the membrane transport mechanisms of L-carnitine in rat aortic endothelium from non-hypertensive WKY and spontaneously hypertensive rats. Furthermore, since (a) the reactivity of aortic rings to the endothelium-dependent vasodilator CGRP was reduced in preparations from SHR compared with WKY rats, (b) L-carnitine supplementation *in vitro* restored CGRP vasodilation to values in vessels from normotensive rats, and (c) CGRP was ineffective in endothelium-denuded rat aortic rings, it is suggested that restoration of a functional endothelium could result from bioavailability of L-carnitine to the aorta endothelium in the spontaneously hypertensive animals.

## References

[1] Melander O (2001) Genetic factors in hypertension–what is known and what does it mean? Blood Press 10: 254–270. 11822531 10.1080/080370501753400575

[2] Mancia G, De Backer G, Dominiczak A, Cifkova R, Fagard R, et al (2007) ESH/ESC 2007 Guidelines for the management of arterial hypertension. Rev Esp Cardiol 60: 968–994. 17915153 10.1157/13109650

[3] Arduini A, Bonomini M, Savica V, Amato A, Zammit V (2008) Carnitine in metabolic disease: potential for pharmacological intervention. Pharmacol Ther 120: 149–156. 18793670 10.1016/j.pharmthera.2008.08.008

[4] Ruggenenti P, Cattaneo D, Loriga G, Ledda F, Motterlini N, et al (2009) Ameliorating hypertension and insulin resistance in subjects at increased cardiovascular risk: effects of acetyl-L-carnitine therapy. Hypertension 54: 567–574. 19620516 10.1161/HYPERTENSIONAHA.109.132522

[5] Rajasekar P, Palanisamy N, Anuradha CV (2007) Increase in nitric oxide and reductions in blood pressure, protein kinase C beta II and oxidative stress by L-carnitine: a study in the fructose-fed hypertensive rat. Clin Exp Hypertens 29: 517–530. 18058477 10.1080/10641960701743998

[6] Furuichi Y, Sugiura T, Kato Y, Shimada Y, Masuda K (2010) OCTN2 is associated with carnitine transport capacity of rat skeletal muscles. Acta Physiol 200: 57–64. 10.1111/j.1748-1716.2010.02101.x 20219053

[7] McCarty MF (2004) A shift in myocardial substrate, improved endothelial function, and diminished sympathetic activity may contribute to the anti-anginal impact of very-low-fat diets. Med Hypotheses 62: 62–71. 14729006 10.1016/s0306-9877(03)00232-9

[8] Miguel-Carrasco JL, Mate A, Monserrat MT, Arias JL, Aramburu O, et al (2008) The role of inflammatory markers in the cardioprotective effect of L-carnitine in L-NAME-induced hypertension. Am J Hypertens 21: 1231–1237. 18787523 10.1038/ajh.2008.271

[9] Gómez-Amores L, Mate A, Miguel-Carrasco JL, Jiménez L, Jos A, et al (2007) L-Carnitine attenuates oxidative stress in hypertensive rats. J Nutr Biochem 18: 533–540. 17142029 10.1016/j.jnutbio.2006.10.004

[10] Sharma S, Aramburo A, Rafikov R, Sun X, Kumar S, et al (2013) L-Carnitine preserves endothelial function in a lamb model of increased pulmonary blood flow. Pediatr Res 74: 39–47. 23628882 10.1038/pr.2013.71 PMC3709010

[11] Volek JS, Judelson DA, Silvestre R, Yamamoto LM, Spiering BA, et al (2008) Effects of carnitine supplementation on flow-mediated dilation and vascular inflammatory responses to a high-fat meal in healthy young adults. Am J Cardiol 102: 1413–1417. 18993165 10.1016/j.amjcard.2008.07.022

[12] Tamai I (2013) Pharmacological and pathophysiological roles of carnitine/organic cation transporters (OCTNs: SLC22A4, SLC22A5 and SLC22A21). Biopharm Drug Dispos 34: 29–44. 22952014 10.1002/bdd.1816

[13] Yabuuchi H, Tamai I, Nezu J, Sakamoto K, Oku A, et al (1999) Novel membrane transporter OCTN1 mediates multispecific, bidirectional, and pH-dependent transport of organic cations. J Pharmacol Exp Ther 289: 768–773. 10215651

[14] Gründemann D, Harlfinger S, Golz S, Geerts A, Lazar A, et al (2005) Discovery of the ergothioneine transporter. Proc Natl Acad Sci USA 102: 5256–5261. 15795384 10.1073/pnas.0408624102 PMC555966

[15] Mo JX, Shi SJ, Zhang Q, Gong T, Sun X, et al (2011) Synthesis, transport and mechanism of a type I prodrug: L-carnitine ester of prednisolone. Mol Pharm 8: 1629–1640. 21854030 10.1021/mp100412z

[16] Tamai I, Ohashi R, Nezu JI, Sai Y, Kobayashi D, et al (2000) Molecular and functional characterization of organic cation/carnitine transporter family in mice. J Biol Chem 275: 40064–40072. 11010964 10.1074/jbc.M005340200

[17] Enomoto A, Wempe MF, Tsuchida H, Shin HJ, Cha SH, et al (2002) Molecular identification of a novel carnitine transporter specific to human testis. Insights into the mechanism of carnitine recognition. J Biol Chem 277: 36262–36271. 12089149 10.1074/jbc.M203883200

[18] Tamai I, Ohashi R, Nezu J, Yabuuchi H, Oku A, et al (1998) Molecular and functional identification of sodium ion-dependent, high affinity human carnitine transporter OCTN2. J Biol Chem 273: 20378–20382. 9685390 10.1074/jbc.273.32.20378

[19] Seth P, Wu X, Huang W, Leibach FH, Ganapathy V (1999) Mutations in novel organic cation transporter (OCTN2), an organic cation/carnitine transporter, with differential effects on the organic cation transport function and the carnitine transport function. J Biol Chem 274: 33388–33392. 10559218 10.1074/jbc.274.47.33388

[20] Wu X, Huang W, Prasad PD, Seth P, Rajan DP, et al (1999) Functional characteristics and tissue distribution pattern of organic cation transporter 2 (OCTN2), an organic cation/carnitine transporter. J Pharmacol Exp Ther 290: 1482–1492. 10454528

[21] Ohashi R, Tamai I, Yabuuchi H, Nezu JI, Oku A, et al (1999) Na^+^-dependent carnitine transport by organic cation transporter (OCTN2): its pharmacological and toxicological relevance. J Pharmacol Exp Ther 291: 778–784. 10525100

[22] Ohashi R, Tamai I, Nezu J, Nikaido H, Hashimoto N, et al (2001) Molecular and physiological evidence for multifunctionality of carnitine/organic cation transporter OCTN2. Mol Pharmacol 59: 358–366. 11160873 10.1124/mol.59.2.358

[23] Okura T, Kato S, Deguchi Y (2013) Functional expression of organic cation/carnitine transporter 2 (OCTN2/SLC22A5) in human brain capillary endothelial cell line hCMEC/D3, a human blood-brain barrier model. Drug Metab Pharmacokinet. doi: 10.2133/dmpk.DMPK-13-RG-058. 10.2133/dmpk.dmpk-13-rg-058 23877104

[24] Herrera MD, Bueno R, De Sotomayor MA, Pérez-Guerrero C, Vázquez CM, et al (2002) Endothelium-dependent vasorelaxation induced by L-carnitine in isolated aorta from normotensive and hypertensive rats. J Pharm Pharmacol 54: 1423–1427. 12396307 10.1211/002235702760345536

[25] Bueno R, Alvarez de Sotomayor M, Perez-Guerrero C, Gomez-Amores L, Vazquez CM, et al (2005) L-carnitine and propionyl-L-carnitine improve endothelial dysfunction in spontaneously hypertensive rats: different participation of NO and COX-products. Life Sci 77: 2082–2097. 15958269 10.1016/j.lfs.2005.01.035

[26] Zambrano S, Blanca AJ, Ruiz-Armenta MV, Miguel-Carrasco JL, Arévalo M, et al (2013) L-Carnitine protects against arterial hypertension-related cardiac fibrosis through modulation of PPAR-γ expression. Biochem Pharmacol 85: 937–944. 23295156 10.1016/j.bcp.2012.12.021

[27] Oza NB, Schwartz JH, Goud HD, Levinsky NG (1990) Rat aortic smooth muscle cells in culture express kallikrein, kininogen, and bradykininase activity. J Clin Invest 85: 597–600. 2298924 10.1172/JCI114479 PMC296465

[28] Guzmán-Gutiérrez E, Westermeier F, Salomón C, González M, Pardo F, et al (2012) Insulin-increased L-arginine transport requires A_2A_ adenosine receptors activation in human umbilical vein endothelium. PLoS One 7: e41705. 22844517 10.1371/journal.pone.0041705 PMC3402464

[29] Livak KJ, Schmittgen TD (2001) Analysis of relative gene expression data using real-time quantitative PCR and the 2-ΔΔCT Method. Methods 25: 402–408. 11846609 10.1006/meth.2001.1262

[30] Kanakasabai S, Pestereva E, Chearwae W, Gupta SK, Ansari S, et al (2012) PPARγ agonists promote oligodendrocyte differentiation of neural stem cells by modulating stemness and differentiation genes. PLoS One 7: e50500. 23185633 10.1371/journal.pone.0050500 PMC3503969

[31] Mels CM, Schutte AE, Erasmus E, Huisman HW, Schutte R, et al (2013) L-carnitine and long-chain acylcarnitines are positively correlated with ambulatory blood pressure in humans: the SABPA study. Lipids 48: 63–73. 23099889 10.1007/s11745-012-3732-8

[32] Tamai I, Yabuuchi H, Nezu J, et al (1997) Cloning and characterization of a novel human pH-dependent organic cation transporter, OCTN1. FEBS Lett 419: 107–111. 9426230 10.1016/s0014-5793(97)01441-5

[33] Srinivas SR, Prasad PD, Umapathy NS, Ganapathy V, Shekhawat PS (2007) Transport of butyryl-L-carnitine, a potential prodrug, via the carnitine transporter OCTN2 and the amino acid transporter ATB(0,+). Am J Physiol 293: G1046–G1053. 10.1152/ajpgi.00233.2007 PMC3583010 17855766

[34] Koepsell H, Lips K, Volk C (2007) Polyspecific organic cation transporters: structure, function, physiological roles, and biopharmaceutical implications. Pharm Res 24: 1227–1251. 17473959 10.1007/s11095-007-9254-z

[35] Koepsell H (2013) The SLC22 family with transporters of organic cations, anions and zwitterions. Mol Aspects Med 34: 413–435. 23506881 10.1016/j.mam.2012.10.010

[36] Devés R, Boyd CA (1998) Transporters for cationic amino acids in animal cells: discovery, structure, and function. Physiol Rev 78: 487–545. 9562037 10.1152/physrev.1998.78.2.487

[37] Mann GE, Yudilevich DL, Sobrevia L (2003) Regulation of amino acid and glucose transporters in endothelial and smooth muscle cells. Physiol Rev 83: 183–252. 12506130 10.1152/physrev.00022.2002

[38] Iwata D, Kato Y, Wakayama T, Sai Y, Kubo Y, et al (2008) Involvement of carnitine/organic cation transporter OCTN2 (SLC22A5) in distribution of its substrate carnitine to the heart. Drug Metab Pharmacokinet 23: 207–215. 18574325 10.2133/dmpk.23.207

[39] Urban TJ, Yang C, Lagpacan LL, Brown C, Castro RA, et al (2007) Functional effects of protein sequence polymorphisms in the organic cation/ergothioneine transporter OCTN1 (SLC22A4). Pharmacogenet Genomics 17: 773–782. 17700366 10.1097/FPC.0b013e3281c6d08e.

[40] Grube M, Meyer zu Schwabedissen HE, Präger D, Haney J, Möritz KU, et al (2006) Uptake of cardiovascular drugs into the human heart: expression, regulation, and function of the carnitine transporter OCTN2 (SLC22A5). Circulation 113: 1114–1122. 16490820 10.1161/CIRCULATIONAHA.105.586107

[41] Foster KA, O'Rourke B, Reibel DK (1985) Altered carnitine metabolism in spontaneously hypertensive rats. Am J Physiol 249: 183–186. 10.1152/ajpendo.1985.249.2.E183 3161337

[42] Shennan DB, Calvert DT, Backwell FR, Boyd CA (1998) Peptide aminonitrogen transport by the lactating rat mammary gland. Biochim Biophys Acta 1373: 252–260. 9733976 10.1016/s0005-2736(98)00107-2

[43] Wu X, Prasad PD, Leibach FH, Ganapathy V (1998) cDNA sequence, transport function, and genomic organization of human OCTN2, a new member of the organic cation transporter family. Biochem Biophys Res Commun 246: 589–595. 9618255 10.1006/bbrc.1998.8669

[44] Aravena C, Beltrán AR, Cornejo M, Torres V, Díaz ES, et al (2012) Potential role of sodium-proton exchangers in the low concentration arsenic trioxide-increased intracellular pH and cell proliferation. PLoS One 7: e51451. 23236503 10.1371/journal.pone.0051451 PMC3516555

